# Stress indicator gene expression profiles, colony dynamics and tissue development of honey bees exposed to sub-lethal doses of imidacloprid in laboratory and field experiments

**DOI:** 10.1371/journal.pone.0171529

**Published:** 2017-02-09

**Authors:** Lina De Smet, Fani Hatjina, Pavlos Ioannidis, Anna Hamamtzoglou, Karel Schoonvaere, Frédéric Francis, Ivan Meeus, Guy Smagghe, Dirk C. de Graaf

**Affiliations:** 1 Laboratory of Molecular Entomology and Bee Pathology, Faculty of Sciences, Ghent University, Ghent, Belgium; 2 Division of Apiculture, Institute of Animal Science, Hellenic Agricultural Organisation ‘DEMETER’, Nea Moudania, Greece; 3 Department of Functional and Evolutionary Entomology, Gembloux Agro-Bio Tech, University of Liège, Liège, Belgium; 4 Department of Crop Protection, Faculty of Bioscience Engineering, Ghent University, Ghent, Belgium; University of Illinois at Urbana-Champaign, UNITED STATES

## Abstract

In this study, different context-dependent effects of imidacloprid exposure on the honey bee response were studied. Honey bees were exposed to different concentrations of imidacloprid during a time period of 40 days. Next to these variables, a laboratory-field comparison was conducted. The influence of the chronic exposure on gene expression levels was determined using an in-house developed microarray targeting different immunity-related and detoxification genes to determine stress-related gene expression changes. Increased levels of the detoxification genes encoding, CYP9Q3 and CYT P450, were detected in imidacloprid-exposed honey bees. The different context-dependent effects of imidacloprid exposure on honey bees were confirmed physiologically by decreased hypopharyngeal gland sizes. Honey bees exposed to imidacloprid in laboratory cages showed a general immunosuppression and no detoxification mechanisms were triggered significantly, while honey bees in-field showed a resilient response with an immune stimulation at later time points. However, the treated colonies had a brood and population decline tendency after the first brood cycle in the field. In conclusion, this study highlighted the different context-dependent effects of imidacloprid exposure on the honey bee response. These findings warn for possible pitfalls concerning the generalization of results based on specific experiments with short exposure times. The increased levels of CYT P450 and CYP9Q3 combined with an immune response reaction can be used as markers for bees which are exposed to pesticides in the field.

## Introduction

Imidacloprid is a neurotoxic insecticide that acts on the nicotinergic acetylcholine receptors of the central nervous system of insects and belongs to the class of chemicals called neonicotinoids [1]. The latter show a high insecticidal activity and are used against a broad range of agricultural pests. Nevertheless, because of their activity against non-target organisms among which pollinating insects, bees in particular, these insecticides are the subjects of debate in Europe and beyond.

Honey bees are exposed to these chemicals throughout the foraging season in several ways [2], though the intoxication mostly does not reach the acute level and has been reported in the context of dust drift and spraying. More common and a much greater effect can be attributed to the sub-lethal exposure of these neonicotinoids. Although it shows no immediate effect on the lifespan of the bees [3], the result of exposure is rather subtle with clear behavioural and physiological consequences for the bees [4, 5]. Many recent studies have demonstrated that low doses of imidacloprid also have sub-lethal effects at colony level [6]. The dynamics of a honey bee colony is the result of a wide range of physiological and behavioural changes such as development of brood and population over time. These variables are predictors for the ability of the colony to survive over winter and to reproduce [7, 8]. Imidacloprid in sub-lethal concentrations caused adverse effects on queen bee fecundity and behaviour with short-term colony functioning as a result [9]. Together with the colony dynamics, the development of particular tissues such as the hypopharyngeal glands (HPGs) of the bees (the glands producing the royal jelly) is considered as a robust indicator of developmental/physiological failure due to pesticide exposure [10, 11]. Development of HPGs starts very early in the life of the adult honey bees, and the glands reach their maximum size and weight when the honey bees are 8–12 days old [12].

Honey bees are primarily exposed to chemicals that beekeepers voluntarily administer in order to control the ectoparasitic mite, *Varroa destructor*, and there are several scientific reports describing the effect of acaricides on bees at the molecular level. Chemicals like thymol, coumaphos and formic acid are able to alter detoxification pathways, immune processes and the expression of some developmental genes [13, 14]. However, the first reports on the molecular effect of fungicides and pesticides on bees, focussed on their larval stage only. The fungicides myclobutanil and chlorothalonil caused elevation of prophenoloxidase-activating enzyme (PPOact) expression, an immune end product, whereas imidacloprid was responsible for the up-regulation of the Hsp70 gene expression [15]. These less pronounced changes following exposure to exogenous chemicals conflicted in a way with the sub-lethal effects that these chemicals may cause. In a recent study on adult bees with an unbiased approach (RNAseq) where bees were chronically exposed to the insecticides fipronil or imidacloprid, no significant impact on detoxifying enzymes was observed, however some immune-related genes were down-regulated [16]. Both above mentioned studies were designed in an laboratory environment: the first used *in vitro* reared larvae [15], the second caged adults [16]. Furthermore, very few studies have been performed comparing results under laboratory and field conditions and using field realistic concentrations of a chemical stressor.

This study compared the respons of caged bees that were chronically exposed to sub-lethal and field realistic concentrations of imidacloprid with bees that received similar concentrations in the field. The expression changes of a set of 109 target genes were screened with an in-house developed colorimetric array, the so-called BeeClinic. This array contained the most important stress indicator genes. The determined effects on gene expression were quantified by real-time PCR. In parallel the changes in the diameter of the acini of the hypopharyngeal glands, the survival rate of the adult bees as well as the brood and population changes of the colonies in the field were studied.

## Materials and methods

### Experimental design

Laboratory and field experiments were performed at the Division of Apiculture of the Hellenic Agricultural Organisation ‘DEMETER’ (Nea Moudania, Greece) with the local *Apis mellifera macedonica* honey bees.

The cage experiments were mainly performed as explained elsewhere [11]. In brief, wooden, mesh-sided cages (10 cm X 10 cm X 10 cm) with a removable metal floor sheet were used. Combs of capped brood from two different colonies were put in an incubator at 34°C and 70% relative humidity (RH). The next day, emerging adult honey bees were collected and transferred to cages (80 bees each). The newly emerged bees from both colonies were homogenously distributed in all control and experimental cages to eliminate possible colony-level effects on the results. Cages were incubated in complete darkness at 28–29°C and 70% (RH). Bees were fed sugar solution (33% w/v) and pollen patty *ad libitum*. Imidacloprid was administered at a concentration of 5 ppb (group C5) and 200 ppb (group C200) in the sugar solution and served as liquid carbohydrate source or it was mixed with pollen pellets (700 g pollen + 300 g of contaminated sugar solution) as the semi-solid protein source (pollen patty). The contaminated pollen patty was prepared by mixing the pollen with the sugar solution containing the correct amount of imidacloprid to ensure the correct imidacloprid concentration in the pollen patty. The 5 ppb and 200 ppb concentrations were chosen as similar to the respective concentrations of imidacloprid found in nectar / pollen / pollen pellets and guttation fluid of seed dressed plants [17–21]. Aliquots were made, kept in the freezer and used every time to prepare the fresh sugar solution for feeding the bees. The negative controls received no imidacloprid in sugar solution or pollen patty (group C0). Three replicates (cages) were used for each imidacloprid concentration and control group. The mortality rates were daily recorded, dead bees were removed and the food was daily renewed.

For the field experiment, 10 colonies were used per group. All colonies received 500 ml of sugar solution twice a week (with or without imidacloprid; same concentrations as above: 5 ppb = group F5; 200 ppb = group F200; no imidacloprid = F0) and 250 g pollen patty (prepared with the corresponding sugar solutions) once a week. Frequent controls ensured that the bees were consuming the food during the next days. They also had the opportunity to forage freely in non contaminated food sources. This procedure mimicked the natural situation, where bees do not only forage in pesticide contaminated food sources. This can result in a dilution of the given contamination in the hive storages, depending on the availability of the non contaminated food sources. The effective concentrations of imidacloprid to which honeybees were exposed were evaluated once by chemical analysis on the prepared sugar solution and pollen patty. In detail, one sample of each concentration prepared for pollen patty and sugar solution was analysed using liquid chromatography coupled to mass spectrometry (HPLC-ESI-MS/MS). The detection limit was 0.45 μg/kg and the limit of quantification was 1.35 μg/kg. Imidacloprid was found in concentrations of 3.1 and 206.7 μg/kg in pollen patty and in concentrations of 5.1 and 176.2 in sugar solution, respectively for the 5 ppb and 200 ppb concentration groups.

### Brood and population changes in the field

All colonies were evaluated for the number of brood cells and adult bees (population) at the start of the experiment. This procedure was repeated after 20 and 40 days. The specific time intervals were chosen in order to include two brood cycles for better understanding the colony development. The brood area and the adult bee population were evaluated by visually assessing the percentage of the comb surfaces densely covered by brood or bees respectively. This is a quick and accurate method used in the field. Each percentage value was then quantified into an actual number of brood cells or bee population based on the fact that each side of the Langstroth frame has about 2024 brood cells and 1144 bees (in particular: each side of the frame has 8.8 x 10 cm^2^ surface area and each 10 cm^2^ contains approximately 230 brood cells or 130 adult bees) [22, 23].

### Hypopharyngeal gland measurements

Samples for the hypopharyngeal glands were collected at day 10 from both laboratory and field experiments. In the field experiment, emerging bees were colour-marked on the spot at the start of the experiment (= day 0), in order to have bees of known age. The hypopharyngeal glands were removed by dissecting the heads in insect saline solution [24] and were stained in Coomassie brilliant blue dye R250 [25] for 5 s. The glands were subsequently photographed on a slide (without cover slip) with a Sony CCD-Iris Hi resolution colour video camera under a Leica MZ6 binocular microscope. The Image Pro-Plus software was used to measure the diameters of the acini (lobes) in two perpendicular directions. The average diameter was calculated based on approximately 300 acini per group.

### Total RNA extraction

For the gene expression profiling, bee samples were collected at two time points at day 10 (the same day as for the glands) and ten days later (= day 20) from both the laboratory and field experiment. In the field experiment, emerging bees were colour-marked on the spot at the start of the experiment (= day 0), in order to have bees of known age. Eight bees per group were collected for each time point and were first shortly anaesthetized by chilling before they were killed by decapitation. Each bee was placed into 1 ml RNALater® (Life Technologies) after separation of the thorax from the abdomen for optimum preservation of the tissues. After incubation overnight at 4°C (to guarantee a thorough penetration of the tissue by RNALater®), samples were frozen at -20°C. They were transported to Ghent University (Ghent, Belgium), maintaining the cold chain, where they were stored at -80°C until further use. Total RNA was isolated from each bee, eight biological replicates from each group, using the RNeasy lipid tissue mini kit (Qiagen) starting from one complete honey bee. The tissues were homogenized by mechanical agitation in a TissueLyser (Precellys) for 90 s at 30 Hz, in the presence of a pair stainless steel beads and 1 ml Qiazol lysis reagent. The total RNA was isolated according to the recommendations of the manufacturer’s protocol, eluting the RNA in a final volume of 50 μl. The concentration of the total RNA was measured using a Nanodrop (Isogen).

### Colorimetric array

The targeted genes with their corresponding probes are listed in S1 Table. The oligonucleotides from the targets available in the study of Johnson et al [26] were selected. New oligonucleotides were designed using the AlleleID7 (Premier Biosoft International). All the probes were synthesized and desalted by Integrated DNA Technologies. The probes were printed in duplicate on nitrocellulose coated glass slides by ArrayIT. The printed arrays were stored at room temperature until use. The cDNA was labelled with digoxigenin (DIG) using the Superscript Direct cDNA Labelling system from Invitrogen as described in De Smet et al. [27]. Briefly, 25 μg of total RNA from each individual bee (8 bees per group) was reverse transcribed into cDNA with anchored oligo(dT)20 primers. The RNA was reverse transcribed using 2 mM dATP, dGTP and dCTP, 1.3 mM dTTP and 0.7 mM alkali stable DIG-dUTP (Roche) which results in DIG-labelled cDNA. The original RNA was degraded by alkaline hydrolysis and the labelled cDNA was purified using the Superscirpt III Direct Purification Module (Invitrogen). Purified single-strand labelled cDNA was eluted from the columns in 70 μl DEPC treated water. The microarrays were pre-hybridized with BlockIT solution for 1 hrs at room temperature. After three washes with 5x SSC (0.15 M NaCl– 0.015 M sodium citrate for 1x SSC) and 0.1% sodium dodecyl sulphate, the arrays were mounted in the cassette. Labelled cDNA corresponding with 3 μg total RNA was mixed with hybridization buffer (final concentration is 25% formamide, 5x SSC and 0.1% SDS) to a final volume of 75 μl and was heated for 1 minute at 65°C to denature the cDNA and snap cooled on ice for 30 sec. The labelled cDNA was then transferred to the array and hybridized overnight at 33.5°C. After hybridization, the arrays were washed twice at 45°C for 5 min with 2x SSC and 0.1% SDS, twice with 0.5x SSC and 0.1% SDS at 45°C for 5 min followed by a 5 min wash at room temperature with 0.5x SSC.

The colorimetric detection is a three-step process. In the first step, membranes were treated with 1% blocking solution (Roche) for 30 min to prevent nonspecific attraction of the antibody to the membrane. In the following step the membranes were incubated with 1500x diluted anti-digoxigenin (Roche) in 1% blocking solution. The antibodies were conjugated to alkaline phosphatase which makes colorimetric development possible. The unbound antibody was washed away in two washing steps with PBS for 15 min after which the slides were equilibrated in TBS buffer for 5 min. In the last step, the membrane carrying the hybridized probe and bound antibody conjugate was reacted with the colorimetric detection reagents NBT (nitro blue tetrazolium salt) and BCIP (5-bromo-4-chloro-3-indolyl phosphate). The NBT/BCIP stock solution (Roche) contained 18.75 mg/ml nitro blue tetrazolium chloride and 9.4 mg/ml 5-bromo-4-chloro-3-indolyl-phosphate, toluidine-salt in 67% DMSO (v/v). The slides were incubated in 15 ml TBS buffer containing 300 μl NBT/BCIP for 30 min. Solution of 18.75 mg/ml nitro blue tetrazolium chloride and 9.4 mg/ml 5-bromo-4-chloro-3-indolyl-phosphate, toluidine-salt in 67% (DMSO) (v/v). Solution of 18.75 mg/ml nitro blue tetrazolium chloride and 9.4 mg/ml 5-bromo-4-chloro-3-indolyl-phosphate, toluidine-salt in 67% (DMSO) (v/v). Solution of 18.75 mg/ml nitro blue tetrazolium chloride and 9.4 mg/ml 5-bromo-4-chloro-3-indolyl-phosphate, toluidine-salt in 67% (DMSO) (v/v). Solution of 18.75 mg/ml nitro blue tetrazolium chloride and 9.4 mg/ml 5-bromo-4-chloro-3-indolyl-phosphate, toluidine-salt in 67% (DMSO) (v/v).The reaction was stopped by washing the slides in distilled water. The arrays were dried by centrifugation. Subsequently, the slides were scanned using the ArrayIT® SpotWare^TM^ colorimetric microarray scanner at 16-bit greyscale depth and 5 μm resolution and saved in TIFF format.

### Data analysis

The inverted TIFF images were processed with Mapix (Innopsys) to ascribe a value to the spot intensity, which was corrected for background intensity. The intensity data were normalized within and between arrays using the Bioconductor package limma using the control reference genes RPL8 and actin. To test for differential expression, the bayesian adjusted t-statistics from the linear models for Microarray data (limma) package was used on the combined dataset [28, 29]. The combined dataset possesses the data originating from 8 biological replicates and the technical repetition was also included. Adjusted p-values were calculated using the method developed by Benjamin and Hochberg [30]. The differential expression was calculated by comparing the expression data from an exposed experiment with its corresponding control experiment. Thus, the exposed cage experiments were compared with the non exposed cage experiments from the same exposure time and the exposed field experiments were compared to the non-exposed field experiments with the corresponding exposure time.

### Quantification of gene expression of key proteins

Using random hexamer primers, 2 μg total RNA was retro-transcribed with the RevertAid H Minus First Strand cDNA Synthesis Kit (Thermo Scientific). Based on the results from the colorimetric microarray some genes were selected and their expression levels were quantified by qPCR. Primers for 10 reference genes and a selection of target genes, based on the colorimetric array screening (S2 Table), were used from literature or newly designed with Primer3 (http://www.ncbi.nlm.nih.gov/tools/primer-blast/) using the default settings.

For each primer pair, amplification efficiency estimates were derived from a standard curve generated from a serial dilution of pooled cDNA (1×,10×, 10^2^×, 10^3^×, 10^4^×, and 10^5^× dilutions). Mean quantification cycle (Cq) values of each tenfold dilution were plotted against the logarithm of the pooled cDNA dilution factor. Only primer sets that amplified with efficiencies between 80 and 120% as calculated by the formula efficiency (E) = −1+10^(−1/slope)^ were used, to reduce any error from different amplification efficiencies in Cq data collections.

For the RT-qPCR assays, the Platinum (R) SYBR (R) Green qPCR Supermix-UDG (Live Technologies) was used. Each 15 μl reaction consisted of 7.5 μl master mix, 0.2 μM forward and 0.2 μM reverse primer (Integrated DNA Technologies) and 0.2 μl cDNA template using the CFX96 Real-Time PCR Detection System (Bio-Rad). The PCR program comprises an activation step of 1 min at 95°C and 40 cycles of a combined denaturation (15 s at 95°C) and annealing (30 s at 60°C) step. At the end of this program, a melt curve is generated by measuring fluorescence after each temperature increase of 0.5°C for 5 sec over a range from 65°C to 95°C to verify the presence of the desired amplicon. All reactions were performed in triplicate. no-template controls, containing DEPC-treated water, were included in each run and no-RT controls were runned for all samples. For the qPCR quantification of the selected target genes, four biological replicates from each group (C0, C5, C200, F0, F5 and F200) were included in the experiment.

Reference gene stability was analysed with the geNorm^PLUS^ algorithm within the qBase^PLUS^ environment (Biogazelle NV) with default settings [31]. The geNorm program generates a stability measure (the M value) for every gene, allowing their ranking according to their expression stability (with the lower value indicating increased gene stability across samples). It also generates a pairwise stability (v) measure to decide the benefit of adding extra reference genes for the normalization.

Differential gene expression of 15 different target genes, detoxification and immunity genes, were determined using qPCR. The primers used for these different genes are given in S2 Table. The differential expression was statistically analysed using qBase^PLUS^, by means of one-way ANOVA. Two-sided significance and correction for multiple testing was applied.

## Results

### Adult bee survival in cages and colony development

**Fig 1:**
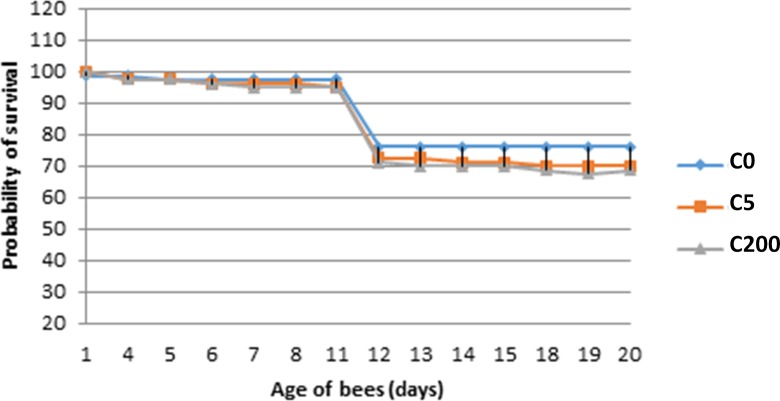
The survival curve of the honey bees in the cage experiment. The survival curve of the honey bees in the cage experiment during 20 days. C0: cage experiment with not treated honey bees; C5: cage experiment with honey bees treated with 5 ppb imidacloprid; C200: cage experiment with honey bees treated with 200 ppb imidacloprid (average of bees in 3 cages per treatment).

**Fig 2:**
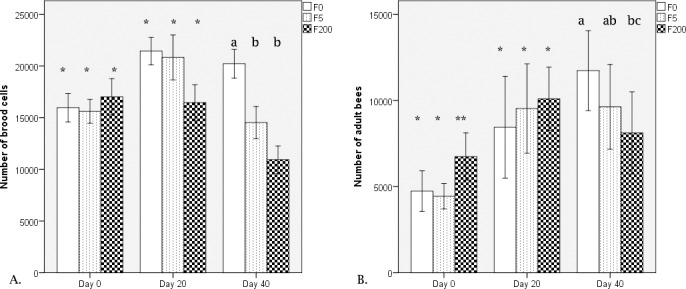
Colony population dynamics. Differences in colony brood and population after 20 and 40 days of treatment with imidacloprid, in the field. Different number of * and different letters above the bars denote significant differences between the groups. F0: field experiment not treated; F5: field experiment treated with 5 ppb imidacloprid; F200: field experiment treated with 200 ppb imidacloprid (average of 10 colonies per treatment).

Honey bees in cages treated with 5 ppb and 200 ppb imidacloprid had a similar survival rate as the non-treated honey bees (Fig 1), confirming that both concentrations were sub-lethal. However, a different pattern appeared in the field when evaluating colony development in terms of number of brood cells and adult bees, where significant differences were detected among the treated groups (Fig 2). In particular, while all groups started with similar number of brood cells, 20 days later, their brood was still equal but after 40 days, the F5 and F200 group were rearing significantly less brood (Repeated Measures Design, Multivariate tests, ‘population’ x ‘treatment’ interaction: Wilks' Lambda F. = 7.520, P<0.001; Fig 2A). The adult bee population showed comparable patterns. The number of adult bees in all groups was similar after 20 days of imidacloprid exposure. However after 40 days of exposure the F200 group increased only with 18% compared to 150% in the control colonies and 104% in the F5 colonies (Repeated Measures Design, Multivariate tests, appeared ‘population’ x ‘treatment’ interaction: Wilks' Lambda F. = 10.537, P<0.001; Fig 2B).

### Hypopharyngeal gland development

**Fig 3:**
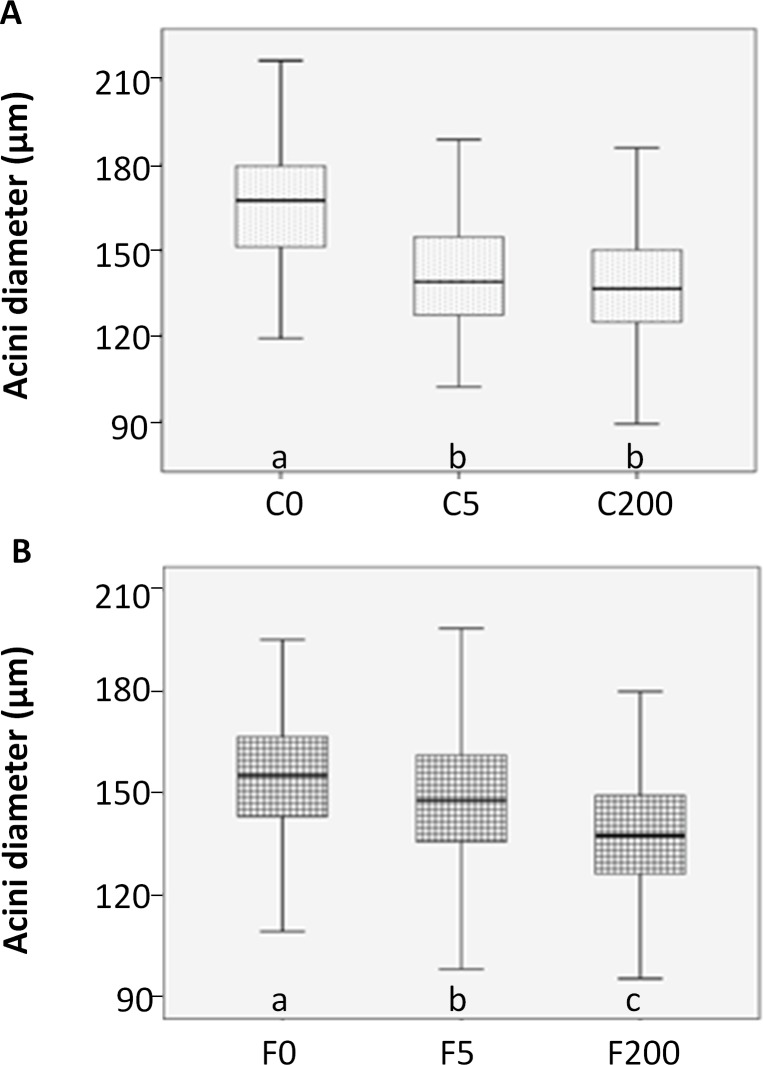
HPG acini diameters. A boxplot from the acini diameter (in micrometer) of the different experimental groups. Different letters below the bars denote significant differences between the groups, P<0.001 for each experimental design. C0: cage experiment not treated; C5: cage experiment treated with 5 ppb; C200: cage experiment treated with 200 ppb; F0: field experiment not treated; F5: field experiment treated with 5 ppb; F200: field experiment treated with 200 ppb.

The acini diameters from honey bees in field were smaller than those from honey bees housed in cages (mean ± SDEV: 155 ± 17 μm for F0; and 166 ± 19 μm for C0). The treatment of the honey bees with imidacloprid decreased the mean acini diameter of the hypopharyngeal glands both in the lab and field experiment (Fig 3). The acini of honey bees in cages treated with 5 and 200 ppb were significantly smaller than those of the untreated group (mean ± SDEV: 141 ± 18 μm for C5 and 139 ± 19 μm for C200; One Way ANOVA, F = 150.167; P<0.001). Under laboratory conditions, no dosis respons effect could be registrated on the decrease of the diameter of the acini (Fig 3A). However, honey bees treated with the same concentrations of imidacloprid in the field reacted differently; the higher the concentration of imidacloprid the smaller the acini diameter of the HPG (One Way ANOVA, F = 71.427; P<0.001). Honey bees treated with 5 ppb had an intermediate gland size (mean ± SDEV: 148 ± 19 μm) while the gland size of bees treated with 200 ppb imidacloprid in the field were comparable with those from treated honey bees in cages (mean ± SDEV: 138 ± 16 μm; Fig 3B).

### Screening for expression effects with colorimetric array

**Fig 4:**
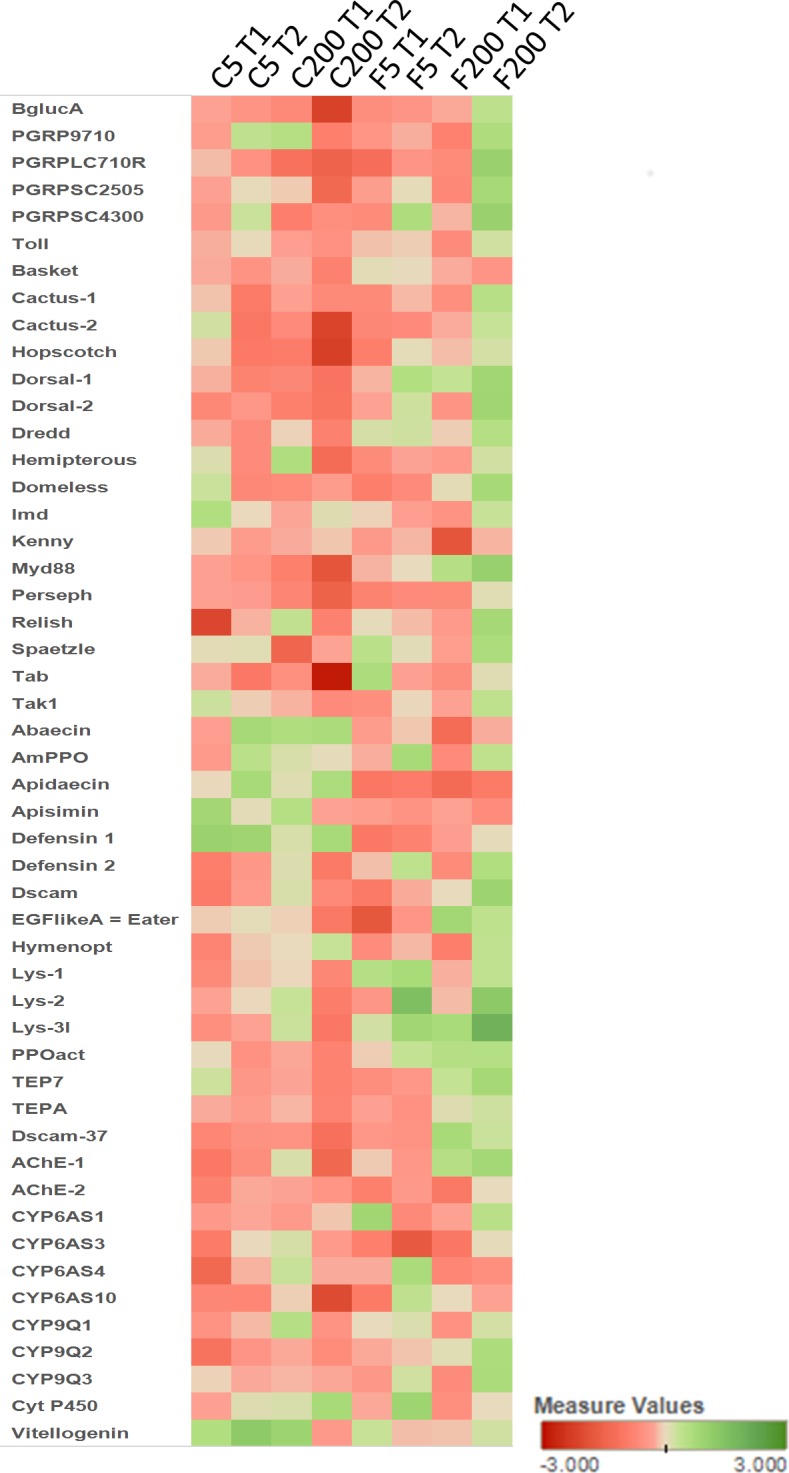
Heat map showing the relative expression profiles of immunity related and detoxification genes. C5 T1 and C5 T2: cage experiment treated with 5 ppb for 10 and 20 days, respectively, compared to the cage control at 10 and 20 days; C200 T1 and C200 T2: cage experiment treated with 200 ppb for 10 and 20 days, respectively, compared to the cage control at 10 and 20 days; F5 T1 and F5 T2 a: field experiment treated with 5 ppb for 10 and 20 days, respectively, compared to the control at 10 and 20 days; F200 T1 and F200 T2: field experiment treated with 200 ppb for 10 and 20 days, respectively, compared to the field control at 10 and 20 days.

The gene expression profiles of the different immunity and detoxification genes are shown in Fig 4 and S3 Table. In general, the treatments suppress the immunity response from honey bees in cages but the response in the field is much less pronounced. Bees which are exposed to higher concentrations show even an immune stimulation at later time points. The same is noticed for the expression profile of different detoxification genes. From these expression profiles, it is clear that bees exposed to imidacloprid react differently depending on their housing conditions.

### Quantification of expression effects for selected genes

For all the primer pairs, melting curve analysis was performed to confirm the specific amplification of each gene and no visible primer-dimer formation. The geNorm algorithm was used to determine the best and most reliable reference genes and to rank all the candidate reference genes according to their stability value for accurate gene expression. Taking into consideration the data obtained from the different treatments, the ranking from the genes from most to least stable is enolase > GADPH > RPS18 > RPL8 > Mlc2 > RPL13a > RPS5 > actin > eIF3 > MGST (S1 Fig). It also generates a pairwise stability measure to decide the benefit of adding extra reference genes for the normalization. The pairwise variation (Vn/Vn+1) was analysed between normalization factors NFn and NFn+1 to determine the optimal number of reference genes required for reliable normalization. Considering the cut off value of 0.15 [32], our data suggest that a minimum of 3 genes is required to normalize the data in the gene expression study (S1 Fig).

**Fig 5:**
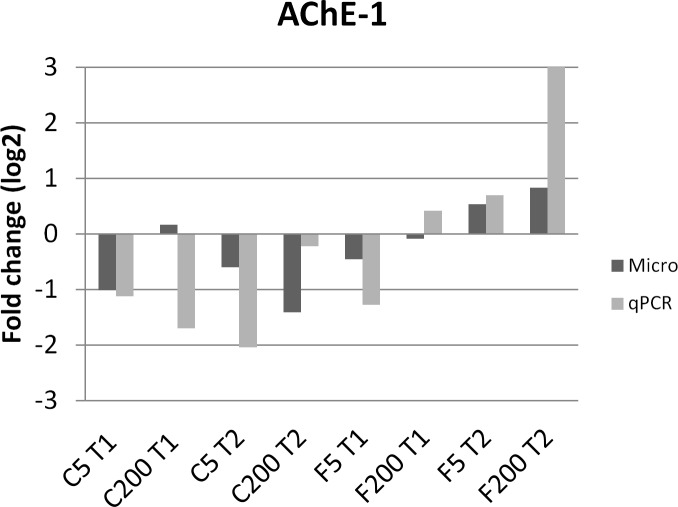
Comparison of the microarray data with the qRT-PCR results. The mean fold changes of mRNA expression of AChE-1 for the different conditions relative to their appropriate control are given on the y-axis. C5 T1 and C5 T2: cage experiment treated with 5 ppb for respectively 10 and 20 days compared to cage experiment non treated for respectively 10 days and 20 days; C200 T1 and C200 T2: cage experiment treated with 200 ppb for respectively 10 and 20 days compared to cage experiment non treated for respectively 10 days and 20 days; F5 T1 and F5 T: field experiment treated with 5 ppb for respectively 10 and 20 days compared to field experiment non treated for respectively 10 days and 20 days; F200 T1 and F200 T2: field experiment treated with 200 ppb for respectively 10 and 20 days compared to field experiment non treated for respectively 10 days and 20 days.

**Fig 6:**
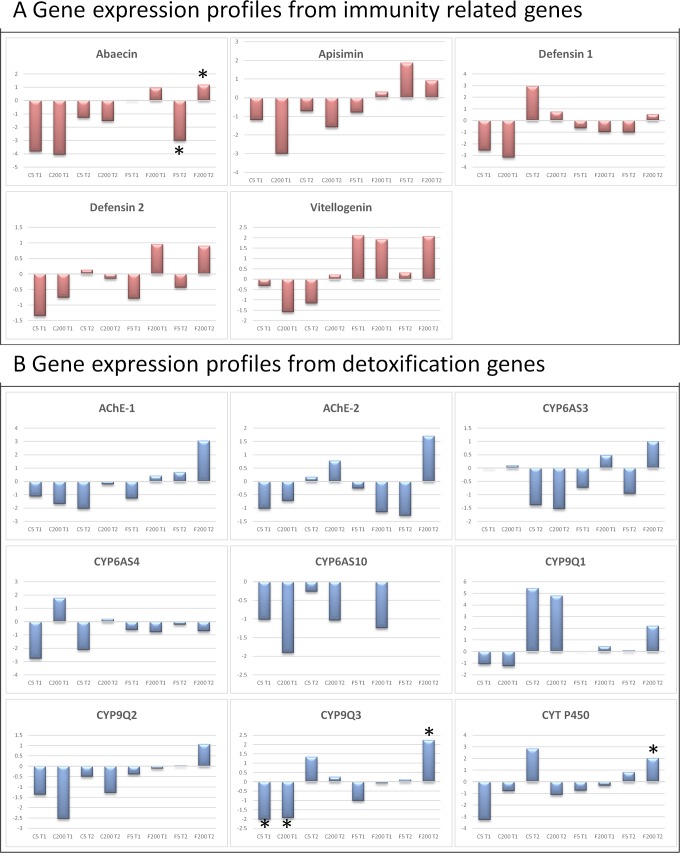
**Expression profile of different immunity related genes (panel A) and detoxification genes (panel B).** The mean fold changes of mRNA expression for the different conditions relative to their appropriate control are given on the y-axis which represents the log2 transformed fold change. *P <0.05, one-way ANOVA. C5 T1 and C5 T2: cage experiment treated with 5 ppb for respectively 10 and 20 days compared to cage experiment non treated for respectively 10 days and 20 days; C200 T1 and C200 T2: cage experiment treated with 200 ppb for respectively 10 and 20 days compared to cage experiment non treated for respectively 10 days and 20 days; F5 T1 and F5 T2 a: field experiment treated with 5 ppb for respectively 10 and 20 days compared to field experiment non treated for respectively 10 days and 20 days; F200 T1 and F200 T2: field experiment treated with 200 ppb for respectively 10 and 20 days compared to field experiment non treated for respectively 10 days and 20 days.

The expression changes for some genes coding for immune end products, vitellogenin and detoxification enzymes were determined using qPCR with gene-specific primers (listed in S2 Table). RNA samples extracted from four biological replicates were used as template for qPCR. According to the results from 18 qPCR sets including the reference genes (enolase, GADPH and RPS18), the trends of expression patterns of the tested genes from microarray analysis were in accordance with the expression levels by qPCR although the altitude of fold change is different due to sensitivity of each technique (Fig 5). The qPCR data confirmed the observation that imidacloprid-exposed honey bees housed in cages react differently compared to exposed honey bees housed in field conditions (Fig 6, S4 Table). Our results suggest that the natural environment of the bees (field condition) is crucial to induce an immunity response and a detoxification process. In the cage experiment, almost all tested immunity-related genes were down-regulated at both time points, with the exception of defensin1 which was up-regulated after 20 days. Under field conditions, the expression profile of the immunity genes was more complex with mostly little or no effect when bees were exposed to 5 ppb of imidacloprid. However, exposure to higher concentrations, 200 ppb, boosted the immunity response. Vitellogenin expression was down-regulated in caged honey bees, while it was up-regulated in bees exposed to imidacloprid under field conditions.

Most detoxification enzymes were down-regulated after 10 days exposure to imidacloprid in caged honey bees with a significant down-regulation of CYP9Q3, while after 20 days some genes, AChE-2, CYP9Q1, CYP9Q3 and CYT P450, were rather up-regulation although these expression changes were not significant. However, under field conditions most detoxification enzymes were up-regulated after 20 days when exposed to higher concentrations, with a significant up-regulation of CYT P450 and CYP9Q3. Some genes were up-regulation after 10 days but at lower levels. Exposure to lower concentrations of imidacloprid under field conditions had little or no effect on the expression levels of the tested detoxification genes.

## Discussion

Different experiments have documented that several neonicotinoid products are toxic to bees. Depending on the amount of exposure to neonicotinoids, the effect on bees can be either lethal or sub-lethal [6, 33–39]. Neonicotinoids have also been implicated, along with fungicides, in either depressing bees’ immune systems or increasing their susceptibility to biological infections [40–42]. Many of these studies were performed under laboratory or semi-field conditions, but none in the open field. The current study clearly showed that the specific reaction of a honey bee on a treatment is more complex and is dependent on several factors.

### The effect of imidacloprid on hypopharyngeal gland and colony development

The size of the HPGs measured as acini diameter is an indication of the gland’s activity and reflects the amount of proteins produced [43]. The different treatments under laboratory versus field conditions induced a different impact on the size of the HPGs. The acini diameters of the control groups (both in the laboratory and the field) were in correspondence with those determined in earlier studies [11, 44–47]. In the cages, the size of the glands of the bees exposed to imidacloprid was significantly smaller than control but independent of the administrated concentration. The effect of imidacloprid, even at very low concentration, reduced the HPG dramatically which do not allow further reduction at higher concentrations. However, in the field the decreasing effect on the size of the glands is directly related to the exposed concentration which may be explained by the expression of detoxification reactions by the honey bees in their natural environment. Clearly the observed effect of the stressor in our study was more apparent and acute in the laboratory than in the field. This can partly be explained by the fact that bees in the field have also access to non-contaminated food which resulted in possible dilution of imidacloprid under field conditions, which does not apply in laboratory studies. Similar results on dose response in the field have been demonstrated in other studies referring to colony development and queen supersedure [9] or to protein content of the bees [48]. It is also possible that the dilution of the contamination is more apparent when the stressor is applied at low concentrations (e.g. 5 ppb) which is in accordance with the observation that the size of the glands are correlated with the exposed concentration in the field and not in the laboratory.

As a result of the reduced HPGs, less brood was produced leading to a smaller population later on. This links a physiological effect at individual level with a performance effect at the colony level. The decreasing gland size follows the same pattern as the decreasing of the brood / population produced later on which support this idea. In the field the optimum amount of royal jelly produced by the HPG glands is essential for maximum development of brood and population. Sub-lethal concentrations of pesticides can lead to colony collapse or dwindling due to homing failure in foraging honey bees [49] which induce an earlier transformation of nursing bees to foragers to compensate for the loss. The size of the glands reduces naturally when the nursing bees start foraging [12] and as pesticides exposure also induce direct gland reduction [11] pesticides may induce earlier transformation of nurses to foragers which also results in a shortened life span [7, 11].

### Detoxification mechanisms upon imidacloprid exposure

A previous study comparing the acetylcholinesterase (AChE) activity in honey bees exposed to imidacloprid reported an increased AChE activity for both in-field and laboratory experiments [50]. This is in correspondence with the expression trends of the recent study. *Apis mellifera* is expressing two isotypes of AChE. In cages, only AChE-2 was responsible for this activity after 20 days of exposure, while in the field AChE-1 and AChE-2 were both up-regulated.

Cytochrome P450 monooxygenases (P450s) catalyse a broad diversity of reactions that contribute to the detoxification of natural and synthetic xenobiotics in insects [51–53]. The CYP6AS clade can metabolize plant secondary compounds found in honey and hive products [54]. Johnson et al. [55] showed no induction of CYP6AS in response to toxic exposure although these findings may partly be explained by the supplied diet. Our results demonstrated that CYP6AS3 was up-regulated only in the field experiment when exposed to higher concentrations. CYP6AS4 was up-regulated after 10 days when the bees were caged and exposed to 200 ppb. These changes were not significant but based on these trends, it is hypothesized that the CYP6AS clade of enzymes may be involved in pesticide detoxification in honey bees. However, a transcriptomic study of imidacloprid-exposed larvae showed elevated transcription levels for a cluster of genes encoding detoxifying P450 enzymes, including CYP6AS and CYP9Q genes [56]. Tau-fluvalinate enhanced CYP9Q3 transcript levels while bifenthrin enhanced CYP9Q2 transcript levels and repressed CYP9Q3 transcript [57]. The recent study showed that all three studied CYP9Q were induced under field conditions when exposed to 200 ppb at later time points. Under cage conditions only CYP9Q1 was up-regulated after 20 days of exposure. As the CYP9Q3 gene and the CYT P450 were significantly up-regulated under field conditions, these enzymes were most probably involved in the detoxification of imidacloprid under natural conditions and may be used as a stress indicators upon pesticide exposure. It is worthwhile to notice that CYP9Q3 was significantly down-regulated in the cage experiment while under field conditions it was up-regulated only after 20 days of exposure. This suggests that the bees need some time to induce the detoxification mechanisms. Moreover the detoxification reaction depended on the housing condition. This is not the first study which reports on a laboratory-field comparison which concluded that bees reacted differently depending on the housing conditions. Individual worker honey bees whose colonies had experienced imidacloprid exposure accumulated significantly increased *Nosema* spore counts after experimental infection [58].

### The immune response is different under field and cage conditions

The influence of imidacloprid exposure on the bees immune-competence was also studied because reduced immuno-competence can lead to increased susceptibility to pathogenic infection, potentially impacting on individual and colony survival [59]. Caged honey bees showed an overall immune suppression upon imidacloprid exposure with an exception of defensin 1, which was up-regulated at later time points. In our field experiments, most of the immunity genes showed an elevated expression level when exposed to higher concentrations. Abaecin was significantly up-regulated when exposed to 200 ppb imidacloprid, while the same gene was significantly down-regulated when exposed to 5 ppb after 20 days of exposure. These results suggest that bees exposed to imidacloprid in field conditions are able to set up an immune reaction while bees housed in artificial cages suppress this reaction. The immune response in the field may also be a result of dilution of contamination or by the detoxification reaction which was induced in the field and not in the cages. The combination of both reactions may result in a more resilient reaction of honey bees in the field. Abaecin was also upregulated in imidacloprid-exposed larvae under field conditions [56].

The vitellogenin expression was the most extreme example of the different reaction capabilities of bees upon imidacloprid exposure depending on their housing condition. Vitellogenin was down-regulated in imidacloprid-exposed bees in cages while those in-field conditions showed an up-regulation. The down-regulation of vitellogenin in cages was in accordance with the study on *in vitro* exposed larvae to imidacloprid [15]. This opposite reaction may be the result of induced detoxification reactions in the field.

In conclusion, this study highlighted the different context-dependent effects of imidacloprid exposure on the honey bee response. These findings together with the possible 'natural dilution' factor of the contamination are very important. Researchers should take these findings into account when designing experiments to study the influence of a specific factor. The study also showed that generalization of the results based on a specific experiment is difficult and should be carefully considered as there is a high complexity of the physiological and behavioural parameters involved, as well as the time and the duration of the exposure of a stressor. The obtained results might explain the differences reported between laboratory and field studies. Moreover, some genes involved in the detoxification of imidacloprid and the immune respons such as abaecin, vitellogenin, CYP9Q3 and CYT P450 may be good candidates to include in a gene expression profiling test to screen for pesticide exposure.

## Supporting information

S1 Fig A: Average expression stability of remaining reference targets. B: Determination of the optimal number of reference targets.
(PDF)

S1 Table Locus names, gene function or marker, oligo sequence and accession numbers for genes on the array. (PDF)

S2 Table List of PCR primers used in this study. The primers are used for qPCR.
(PDF)

S3 Table Overview of the microarray data. (XLSX)

S4 Table Statistical analysis of the qPCR expression data. (XLSX)
