# Supplementary material for: Stress indicator gene expression profiles, colony dynamics and tissue development of honey bees exposed to sub-lethal doses of imidacloprid in laboratory and field experiments
Source: PLoS One. 2017 Feb 9;12(2):e0171529. doi: 10.1371/journal.pone.0171529 (PMC5300173; doi:10.1371/journal.pone.0171529)
Supplement: S1 Fig — A: Average expression stability of remaining reference targets. B: Determination of the optimal number of reference targets. (PDF) [file pone.0171529.s001.pdf]

A

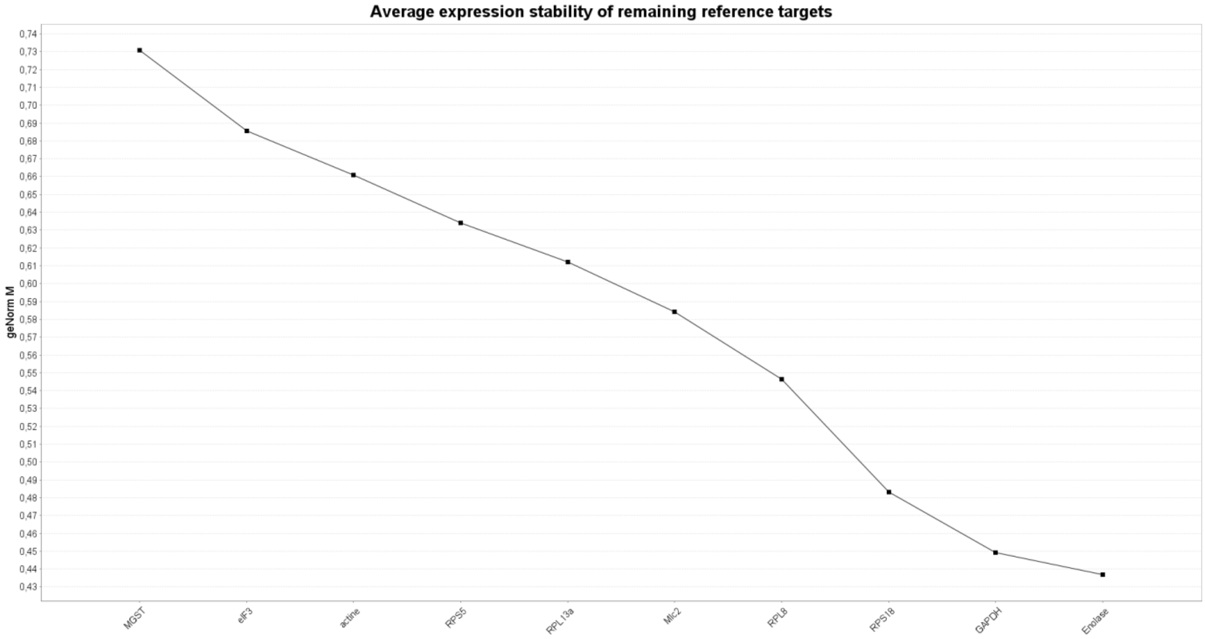

B

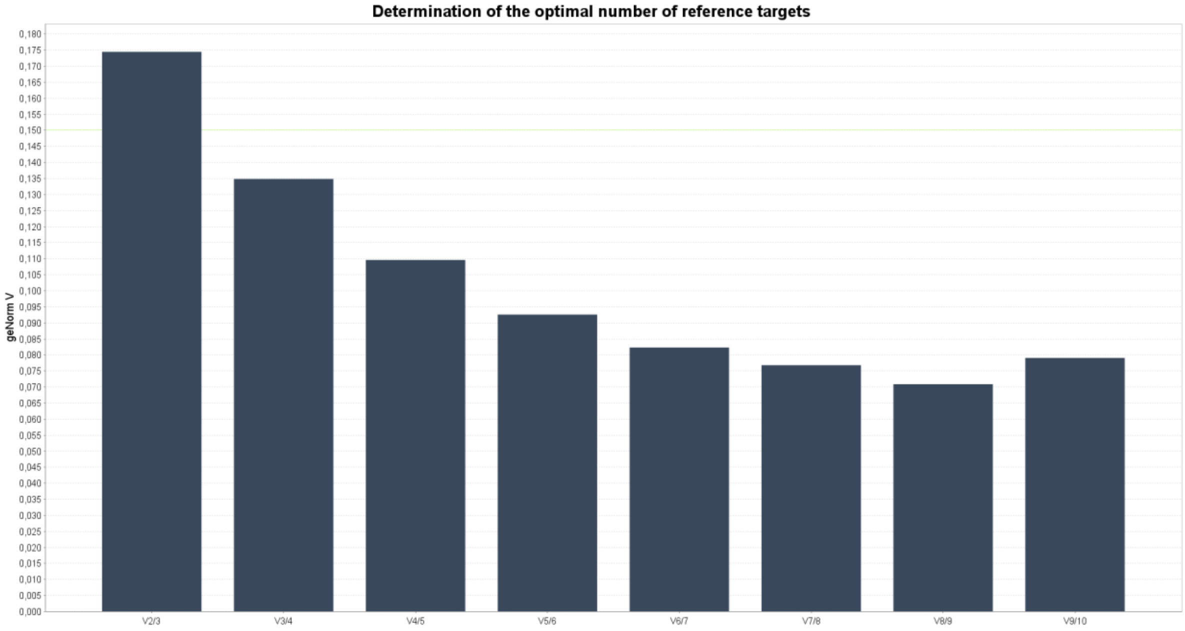

Figure S1 A: Average expression stability of remaining reference targets. B: Determination of the optimal number of reference targets.
