# Supplementary material for: Stress indicator gene expression profiles, colony dynamics and tissue development of honey bees exposed to sub-lethal doses of imidacloprid in laboratory and field experiments
Source: PLoS One. 2017 Feb 9;12(2):e0171529. doi: 10.1371/journal.pone.0171529 (PMC5300173; doi:10.1371/journal.pone.0171529)
Supplement: S1 Table — (PDF) [file pone.0171529.s002.pdf]

S1 Table: Locus names, gene function or marker, oligo sequence and accession numbers for genes on the array.

Locus	Function/Marker	Sequence	Gene ID
BglucA	Immunity-R	GAGTCTATTCCTGGTATTTGTGAAACTGGTTGGAAAAATTATGCTCAGACAAGAACAAGAGGAGTGGCT	XM_006557682
PGRP9710	Immunity-R	GCTACGCTAAGTCCTGGATTTGAACTTTATAAACAGATTCAAAATTGGCCTGAATGGGTTTCTACTCCA	NM_001163715
PGRPLC710R	Immunity-R	GGAAGGAGATTGGATACCTGATTTACCTGTACCAACGGTATTTCAACAACATCAAGAAGTGACAACCGC	XM_006565502-7
PGRPSC2505	Immunity-R	ATGGGGTAGCAATTGGGAAGATACAAAGCAATTATACGTTACTTGGCCATCGACAAACAACTCGCACTT	XM_006564043
PGRPSC4300	Immunity-R	CCTTACGTAGTCGTTCATCACGGAGGGATAATACAATACTGTTTCGATGTGAAAACGTGCTCAGCCATC	XM_001121036, XM_006564054
Toll	Immunity-S	GAGCATGAATACATATGTAAAATGGGGTGATCCTTGGTTTTGGGACAAATTGCGATATGCGTTACCACA	XM_006562720
Basket	Immunity-S/N	GAAAGAGCTGATCTACCAGGAAGTGATGGAATACGAGACGAGCCATAATCCCACGACAGTAGCTCAAAC	XM_006557236-45
Cactus-1	Immunity-S	GAACGACACCAATGGAAAGCGATGTTCCAGAGAATGAGCAACAGAAGTCCGAAAAACAACATAGTTCGG	XM_006567107-8
Cactus-2	Immunity-S	GCCTCCGCAAAAGCACTTACCGATCCTTTAACCTCTATAGAAAGAAATTATCTTCTCCCTGGAAAAAAA	XM_394485
Hopscotch	Immunity-S/NS	GGAACAGCAGAAGATTGTGGATCTGGAAGTAGCAATGGTTCTTTAGGTCAAATGCGAGGAATTTATGAA	XM_001121783, 88-90
Dorsal-1	Immunity-S	AGGCCACAAGTATCGCCAGATCGTACATCGCCAATGGAGTACAGATTATACAATCCAGCACTTATTCAA	XM_006567002
Dorsal-2	Immunity-S	CTGGCTTTGAATTCAAAAGGGATCTCAATTCCGTTGATCTAAATGCAGTGAGGCTGTGCTTCCAAGTGT	XM_006565455, XM_395180
Dredd	Immunity-S	GCAGTAGGGGAAAAAAAAGGAGTACTAAATGGAATAAATTCCGTTGCTCAATTACCCGAA	XM_006570913
Hemipterous	Immunity-S	AGTGGACGTTATTCTCCATTTCTTCAACAACGATTAGGACAAACTCCATCTGTGCCTCAAACTCATCCA	XM_006557980
Domeless	Immunity-S	ACAACACTTGAAGCTGCACCAACAACTCCACCACAGGATGTAAGAATTATTAATGTGACAAATACGACAATGT	XM_003251652
Imd	Immunity-S	AGCGATGCAATACCTGTCACGGGCGTTAATTATCCTACCAAAGAACAACATCTGAATGATACAAAAAGT	XM_006570166
Kenny	Immunity-S	CGACCAAATTGTTGAGTATCAATTCCAAATTGTATGCTGAACCAGAAAGCCACTTACAAAGTCTTGGTT	XM_001120619, XM_006565050
Myd88	Immunity-S	TCTAGACCAAATATATCAGCAAATAGACGTGTACTTCGGACGACGCGAGCTTTAAAAGAAATTCGAT	XM_006560439-41
Perseph	Immunity-S	GAAGCATGTATTACTGACAACAATTCGCCAGGCATTTGTAAACTTCTTCAGCAATGTCCTTCGGTTTATGAA	XM_001120043
Relish	Immunity-S	AAGAACTGACATTGAAGCATACAATGATGGATGGATGCCACTACATCTTGCAGCAAAAGCAGGGTCATA	XM_006562219-21
Spaetzle	Immunity-S	AGCAATGAACAAAGATAATGAATGGAAATATGTTGCGAATCAAGAAAATTTTAAACAGGGTATACGAGT	XM_003250627, XM_006565533-34
Tab	Immunity-S	CGGGCCTTCTTGGGTCTGTAGAATGTGCACGTTTGATAATCATCCGTTGATGAATAAATGCGAGCAATG	XM_001122664, XM_006565777
Tak1	Immunity-S	TTGAAGAACACAAACAACTTGCGCAAGAATATTTGAAAGTTCAAACGGAAATAGCGCTCTTGGGTCAAC	XM_006572294, XM_397248
Abaecin	Immunity-E	CGCCACGATATGCGCAGCATTCGCGTATGTACCATTACCAAATGTACCACAACCAGGACGTCGTCCGTT	XM_006569641
AmPPO	Immunity-E	TTTGTGGATGTGGATGGCCGCAACATATGCTTATACCAAAAGGAAATAAAGAAGGTTTTGCAATGGAAC	KC766703-6
Apidaec	Immunity-E	AAGCTGAACTGGAAGCTGAACCCGGTAATAATCGGCCAGTATATATTTCACAACCAAGACCACCTCATC	XM_006572699
Apisimin	Immunity-E	CCAACGTGTCAGCAGTACTCCTAGCTCAAACTTTAGTTAATATCCTGCAAATTCTTATCGACGCTAA	NM_001011582
Defensin2	Immunity-E	TGAGCATTAATCATTCAGCTTGCGCTATCAGATGTTTAGCTCAACGACGTAAAGGCGGTAGTTGCAGAA	NM_001011638
Defensin1	Immunity-E	ACGTGCCGACAGACATAGAAGAGTAACTTGTGACCTTCTCTCATTCAAAGGACAAGTTAATGACAGTGC	NM_001011616
Dscam	Immunity-E	GAGCAGGCAGGACGTTAATATCATAACTAATAAGAGGGTCAGCTTCTTGAGCATCGACAGCGTTGC	AY686596
EGFlikeA = Eater	Immunity-E/CCD	GTTTCTCTCGAATCACGTGACGATAGGTGGAATAGCTATGGGTATCGCTATGGTTATAACCGGTAGCGC	XM_006561053
Hymenopt	Immunity-E/CCD	TGGACAACCTAGTCGACAGCATGCAGGATTCGAGTTTGGGAAAGAATATAAGAATGGTTTCATCAAAGG	NM_001011615
Lys-1	Immunity-E	AGCCGTTGCCGAATATTGGAAACTGCAAACGACGAAAACGATGGTTGGAAATATTATTGGAATTGGAGC	KC766334
Lys-2	Immunity-E	CAACAACTCCAACAAGCTAGAATCTCAAGGAGCGACATAAGCAGTTGGATCTGTTTGATGCAGAGCG	XM_001120136
Lys-3I	Immunity-E	ACAATTGGTTGTGATGAATCTGTTTGCGGACCATTTCGTATAACATGGAATTATTGGGCTGATGCCGGT	XM_393161, XM_003693393
PPOact	Immunity-E	GCCATTATTGCACCAACTTGAAAACGGTAGATGGGTGAACATCGGGATAGTGTCTTGGGGAATC	XM_001121888
TEP7	Immunity-E	TCATTTGATGAAAGTCCTATGAATTCCTCTTTTGTTGTCAATAACGCCGAGCTAGCAATTCCTGATGGA	XM_006565440-1
TEPA	Immunity-E	GCCATTATTGCACCAACTTGAAAACGGTAGATGGGTGAACATCGGGATAGTGTCTTGGGGAATC	XM_001121888
RPL8	Reference gene	TGGTGGACGTATTGATAAACCAATTCTCAAAGCTGGTCGTGCATATCACAAATACAAGGCTAAGAGGAA	XM_393671
Actine	Reference gene	ACTTGACTTTGAACAGGAAATGGCAACTGCTGCATCATCCTCAAGCTTGGAAAAGAGCTATGAACTTCC	XM_006611156-7
Dscam-37	Immune end product	CGAGAGCCACCGGATATCCGTGATCACTAAGAAAAGGAACAGCCTGCTGTCCATCGATTCGGCCAGCCC	XM_006567065, XM_006567071
Am52C8	Reference gene	GAGTGTCGATTCAAAGTCACGCAAAAGCCAAGAACACCATGATCAAAGCTCGACAATACAGGTTGAACC	NM_001110764

S1 Table: Locus names, gene function or marker, oligo sequence and accession numbers for genes on the array.

KBV	Pathogen	TAATGAGGCTCTCATGAGGGGATGTGGCGAACAGATAGTAAATTTAAGACCCTTACTTCGCACATTCCG	AY275710.1
DWV	Pathogen	CTGCAACATCGGAATCTAACCCAGGACCAAAATTCTTATAATCTCCTGTCACGATGTGAGTGCCAWACT	AJ489744
BQCV	Pathogen	TTCCGAATTCGCTCTAAACGAAGATGACAAACATGGAGCATAGTACGGTATTTGGAACTCTGCGACTCC	AF183905
ABPV	Pathogen	AATTTGCATTGAAGCATCTATGGTATTGATAGAATCTGCAGATGTTACAGGTGGTTGAAACACTTGCGT	AF150629.1
Family ABPV	Pathogen	TCATCGGCTCGGAGCATGGATGATA CGCACAGTATTATTCAGTTTTTACAACGCCCCGTACTCAT	AF150629, AY275710, EU224280
SBV	Pathogen	AACACTCATGTAATAAAGTATACGCCTCTATCTCGTTTACGCCCTCAACATGCTCCATAGTCCACCGCA	AF092924
Ascosphaera apis	Pathogen	CAAAAATGGCCCACTAGTAACGATACATTCAATTGTCCACGTTCAATTAAGCAACAAGGACTTCTTACA	GQ867785
Nosema apis	Pathogen	GCATGATAGTGAGGCTCTATCACTCCGCTGAACTGAGTTGTGTTCTTAAATGGTGTATTGAACCAGGCG	U97150
Nosema cerana	Pathogen	CGCCTGGTTCAATACACCATTTAAGAACACAACTCAGTTCAGCGGAGTGATAGAGCCTCACTATCATGC	U26533.1
CBPV	Pathogen	TACCTATTCCTATATGGGGGAACACAGCAGCCATCGATAGATAAGTCGTCATACGCCCTATCATG	EU122230
SBPV	Pathogen	AGAGTCACTTTAGTTATATTATATAGGTTTATTCGTTTTAAAAGTATAGGATATATCACCACTAGTTGCGTAGCT	EU035616
BSRV	Pathogen	C TTAATGAAGCAATGTAAAGATTTGAACCTTAGTAAGGGAAACATGGAAATCATCGCCCGAAATT	JF423195
LSV	Pathogen	CTCATTTCTTCATGTCAGTGTGTGAGCATGATGAGTCAATACCTACGGTGTTCCATG	HQ871931, HQ888865
APLV	Pathogen	CACATGAACAAAGGAATATCTGTAGCACTGACACGACAAATAATTGACCGATTGATGAGACATTT	HQ871932
Macula like virus	Pathogen	CTACTCCTTCTCTGGTCGCTACTCCTCTATCTCGTGACCCTAGCCTTAAGATCCCTTTTCAGTTC	HQ916350
Apocephalus borealis	*Pathogen*	*AAGAATAACTTCAC ACGTCTACTTATTACCCCTTCACAAATAAGTTCATCT*	*JF798506*
Crithidia sp.	Pathogen	TCACGAACAAGGTGAAGGCCGAGGGCCACGTGAAGGGTGGCGCGAAGAAG	AB716357
Acarapis woodi	Pathogen	ATCAATTCCATCCTCAGTTATTTCTAATGATCAAATTTATAATTCTCTAGTAACTAGACATGC	HF945444.1
Vitellogenin	Nutritional stress/*Varroa* infestation/CCD	GATGCACATTTCAAGGACGTGAAATTGTCCGACTTTGGATTCAGCACTGAGGATATTTTGGACACTGCC	NM_001011578
Transferrin	Nutritional stress/*Varroa* infestation	AACGCAAAATACTTAGACGTGATTGAAAGAAACTCTGGAGCCACGGACAAGATCATTCGCTGGTGTACG	AY336529
Cytochrome P45 monooxygenase (Cyp4g11)	Nutritional stress/*Varroa* infestation	CTTCCGCGTAAGATCCGACGTCAAGGAATCCGAGTTCAGACTACAAGCCGACATTATTTTGAAAAGAGC	XM_006559340-1
Juvenile hormone esterase	Nutritional stress/*Varroa* infestation	TGGACTCAAACAGAAAATTCTTTGGAGAAAGCCAAACAAGTAGGAGCTTTTATGGGTTGCCCTACACGT	XM_006563874
Thioredoxin reductase-1	Nutritional stress	GTGATCAGAGAGTACTTGGCATGCATTTTATTGGTCCTAATGCTGGTGAAGTGATTCAAGGTTTTGCTG	XM_006563201
Alpha-glucosidase (Hbg1)	Nutritional stress	CCGAGACTTACGAATTGGTAAAAGAATGGCGAGATTTTGTGGACAACTATGCAGAAGAAAATAAGCGGG	XM_006560808
Malvolio	Nutritional stress	ACCATTCTTGGCCTTATTATGCAAAGATTAAGTGCAAGACTTGGTGTGGTAACAGGTTTGCATTTGGCA	XM_006563051-2, XM_623943
Painless	Nutritional stress	GCACTGGCTTTCTTCATTCTCTTTAAAGATGGCGGTAATGAAAATTTTCCAGATCCTGGGCACTCGTTA	XM_001122160, XM_006562454
AChE-1	Pesticide exposure	CGAACACCGAGGAAGGTTTCTACTTTATCATTTATTATCTGACCGAGTTGTTCCACATCGACGGGTCCG	XM_006570223.1
AChE-2	Pesticide exposure	CATCGTATTCGAGAGACGAACCAAAATACTTCATCTTCGATGCGGAGAAGACTGGACTTGGTAAAGGGC	NM_001040230.1
CYP6AS1	Pesticide exposure	TTTGTAAAGAATTTTTCCCACCTTTGTACGAAATAGTCGGTAGTATATTTACATTGAAAG	XM_623424.4
CYP6AS3	Pesticide exposure	TTGGCCAGAGATTTCACCAGGCTGTATATGCCATTCTTGTTCGATATAATTGGCGGTTACTTACAATCG	XM_006565072.1
CYP6AS4	Pesticide exposure	AGTTTACGAGGAATTTACTTCGAGATTTCTTTCCACGTTTCTACAATTTGCTTGGCTTCGTATTACCGT	XM_395671.4
CYP6AS10	Pesticide exposure	TCGTGATCCTGATATCTATCCAAATCCGGAGGTGTTTGATATTAATCGATTCAGCAAGGAAGCAGAAGC	XM_001121037.3
CYP9Q1	Pesticide exposure	CCGTCTGGCCAGATTGTTCAATTTGAAGATCCTGCCCGTTCACATCGAGAAGTTTTTCCAC	XM_006562301.1
CYP9Q2	Pesticide exposure	CAACTGATGATGGATACGAGGAACAAGAAGGAATCGGGTAAGAAGAATCTGACCGTTCAGAACATGGCC	XM_3920005
CYP9Q3	Pesticide exposure	AACATGGGCTCGTTGGTCAGACAAAAGTCCAACTTGCACGACGTGATAGATCGGACGTACAACCTCGAC	XM_006562300.1

S1 Table: Locus names, gene function or marker, oligo sequence and accession numbers for genes on the array.

Prophenoloxidase	*Varroa* infestation	TTTGTGGATGTGGATGGCCGCAACATATGCTTATACCAAAAGGAAATAAAGAAGGTTTTGCAATGGAAC	NM_001011627
Superoxide dismutase	*Varroa* infestation	TGGTGCACATTTCAACCCATTAGGAAAGGATCATGGTGGACCTGATTCAGATATACGTCATGTTGGAGA	AY329355
Sugarless	*Varroa* infestation	TTTTCCACGGATATAGAAACTGCAATCCAGGAAGCGGATCTAATCTTCATTTCGGTGAATACCCCGACG	XM_006570933-4
Poly U binding factor 68kD	*Varroa* infestation	GGAAATAGTACAATGATTCCTCCTGAAAGAGATGAGGAGAGAACTCCCACATCAAGAGGAATTGCAGGA	XM_393194
Stretchin-Mlck	*Varroa* infestation	TTTTGCCAGCAAATGTCATTCGCAGTGATTGTTCTATAGGAGAATTGACACCTTTGCAAATTGCGGTTC	XM_006567451
Hexamerin 70a	*Varroa* infestation	TCCAGTTGTTCTTATATGTTAGTCCAGTCTCTTCGGAATACAATCAGTACAATTCGAGAATATGG	NM_001110764
Hemolectin	*Varroa* infestation	AAACACCTCTGGTTGCTGTCCAAAATTTATGACTATTTGTGATCCTCAGACTTGTCCTTCAGCACCATC	XM_006564518-20
Dopamine receptor, D1	*Varroa* infestation	ATTGAATCTCTTCCATCGTCTACATCACCGATTTGCTAAGTGTAGTACGTTCCGATCAGGGAGAAACGA	XM_006563320-22
Pheromone biosynthesis-activating neuropeptide	*Varroa* infestation	GGACACCATCTCCCAGACTTGGACGTCAGTTGCACAATATTGTTGATAAACCTAGACAAAATTTTAACG	NM_001110712
Glucose dehydrogenase	*Varroa* infestation	GCTGTAACTGTCCATTAAACCCATCGACTGGACCAACACTGGCATCAACATGCGGTGGTTCGTCCTTT	XM_003250181, XM_006563266-8
Hairy	Nosemosis	ACGAGCCCGCATTAACAACTGCTTAAACGATCTGAAGACCCTCATACTAGATGCCATGAAAAAAGA	XM_393948
Armadillo	Nosemosis	CAAGGTTATGACCAGATACCAGTAGATTCGATGCAAGGGTTGGAAATAGGTGGAGGGTCGACGTATG	XM_006557798-801
α Mannosidase I	Nosemosis	AGGGCAAGCTCCTCTACGTGTCAGACTTGAAATACGACAGGCTGGAGCACAAAATGGGTCATC	XM_006562018
Na pump α-subunit	Nosemosis	GACGACCCGAACTTCCCTGTAGACGGACTCCGCTTTGTGGGCCTCATGTCTATGATCGAC	XM_006564159-66
Facilitated trehalose transporter Tret1-like	Nosemosis	CATCGTGGCTTTTTGCGGAATCATAATTCCTACGACAGCATTAATTGCGTTGCTCCTGATTCCAGAGAG	XM_006557804
Trehalose transporter 1	Nosemosis	CTGCATTTGGTTGGATTCCATTGATGAGTCTGATCGTTTACGTGATCGGGTTCTCATTTGGTTTTGGTC	XM_006570576-81
Catalase	Nosemosis	TCGAATGTTCACTTACGGTGATGCTCATAGACATCGTCTTGGCCCAAACAATCTGCAATTACCAGTGAA	NM_001178069
Corticotropin releasing hormone binding protein	Nosemosis	ACAAGCTTAACTGAGCCATATACGTTACGAAATTATGGACGACGCATAAATTGCACTTATGTCGCGCTC	NM_001012615
Sluggish A	Nosemosis	GCGAAACGTTCCAAGTACCAGACATTAAAACAGGAAAGATGGTTAGGCTTATGTCACAGCTTACGTCCA	XM_006558616
Aubergine	*Nosema* Resistance/QTL	ATGGGTCGTATGAGACGCGATGTCGAAAGATTCATACAAACGCTTAAAGAATCTGCATCTGGAATGGGT	NM_001165906
Futsch	*Varroa* Resistance/QTL	ACGGCAACTCGTATTACACCTCGTTGGAATTTTTCAAAAGGGTACGGGCTAGATATTACGTGTTCAGCG	XM_006560850
Foxo	*Varroa* Resistance/QTL	TGATGCAGAATATTCCATATTTCAGGGAGAAGGGAGAAAGCAACAGTAGCGCCGGATGGAA	XM_001122804
TOR	Aging	GCTGCATTAAAAGGTAAAAGATCTGATGCTCAGGGAATGAGTGCTAGCAGTAATCAAGAGCAGAGCGAT	XM_006566642
IRS	Aging	CAATCCGGTAGTAGCCCTAAGTTAATTTCATCGCCTATGCAAGAAGAAAGTGTGCAATCGAATGGACCG	XM_006565041-44
InR-2 (daf-2)	Aging	CATGTTGCTGAAACACATCGACTCGGACAGTTTTCAAGAGGTTAGCTTCTATCACAGCCCGG	XM_006568694-5
dPGC-1/spargel	Aging	AGAAGGATAGCGGTTTGGAATCAGGAGAAGCAAGTGATAACAGTGAAGAACAAGCAGGAGTTACAACAG	XM_006571743
dSir2	Aging	GGATCGTCATTGAAGGTCCGGCCAGTAGCATTAATACCTTCTTCTATTCCATCTCATGTTCCACAAATT	XM_006569336
Rpd3	Aging	ACAGTTTCTTTTCATAAATATGGTGAATATTTTCCTGGCACTGGTGACCTCCGTGATATTGGTGCGGGA	XM_394976
AmFor		TGCGATTTCGTGGTAAAGTTGTTCAAAACGTTCAAGGATAGGAAGTATCTTTACATGCTGATGGAAGCC	NM_001011581
EGFR	Development	GTATGGCCCAATATTGTTAATGAATTGACAAAAGATAATAATGAAGAATTATTAGACGTTAACAAATGG	XM_006572034
PKA_C1		TTACCAAAAGGTATGGGAATTTGAAGGCTGGTGTAAACGATATCAAGGGTCACAAGTGGTTCGCCAGTA	XM_393285.5
