# Supplementary material for: Stress indicator gene expression profiles, colony dynamics and tissue development of honey bees exposed to sub-lethal doses of imidacloprid in laboratory and field experiments
Source: PLoS One. 2017 Feb 9;12(2):e0171529. doi: 10.1371/journal.pone.0171529 (PMC5300173; doi:10.1371/journal.pone.0171529)
Supplement: S2 Table — The primers are used for qPCR. (PDF) [file pone.0171529.s003.pdf]

S2 Table: List of PCR primers used in this study. The primers are used for qPCR.

Target gene	**Sequence (5'-3')**	**Reference**
REFERENCE GENES		
Actin	F: TGCCAACACTGTCCTTTCTG<br>R: AGAATTGACCCACCAATCCA	[1]
Enolase	F: GGTGATGAAGGTGGTTTTGC<br>R: GATGCAGCAACATCCATACC	[2]
GAPDH	F: GATGCACCCATGTTTGTTTG<br>R: TTTGCAGAAGGTGCATCAAC	[2]
eIF3-S8	F: TGAGTGTCTGCTATGGATTGC<br>R: TCGCGGCTCGTGGTA	[3]
MGST	F: TTGCTCTGTAAGGTTGTTTTGC<br>R: TGTCTGGTTAACTACAAATCCTCCTG	[4]
RP49	F: CGTCATATGTTGCCAACT<br>R: TTGAGCACGTTCAACAAT	[5]
RPL8	F: TGGATGTTCAACAGGGTTCATA<br>R: CTGGTGGTGGACGTATTGATAA	[6]
RPL13a	F: TGGCCATTTACTTGGTCGTT<br>R: GAGCACGGAAATGAAATGGT	[2]
RPS5	F: AATTATTTGGTCGCTGGAATT<br>R: TAACGTCCAGCAGAATGTGGTA	[6]
RPS18	F: GATTCCCGATTGGTTTTTG<br>R: CCCAATAATGACGCAAACCT	[2]
TBP-af	F: TTGGTTTCATTAGCTGCA<br>R: ACTGCGGGAGTCAAATCT	[7]
Target genes		
AChE-1	F: AGTTGGGCGAGATATGGTTG<br>R: GGAAAAATAGAGCGCGTGAG	This study
AChE-2	F: AACCGGCTTAAGGATTCGAT<br>R: CCCTCTCCCCTTCAATCTTC	This study
CYP6AS1	F: GCGACCAATGCGAATGAAAC<br>R: TCACGGCATTCCACCATTTC	This study
CYP6AS3	F: TCGAAAGGGACGAGGATATG<br>R: AGTCATGGGATGCCTACTGG	This study
CYP6AS4	F: GGCTGGATTTGAAACGTCAT<br>R: CGCGTGGAATTCTTTCATTT	This study
CYP6AS10	F: TTTCTCAAAATTCGCCCCATC<br>R: CGGGGACAATTTCGTTCTTA	This study
CYP9Q1	F: GTTCACGTCGAGCAAGATCA<br>R: TCGTCAACACGCTCTTCAAC	This study
CYP9Q2	F: CCTGATCAAGAGCATCACGA<br>R: GATCTTGCTCGAGGTGAAGG	This study
CYP9Q3	F: GTAGCCATTCACGCGTTCAC<br>R: GTCTCGTCGATCTCCTGCTG	This study
CYP450	F: GGCCGGTCAAAATGGTGTTC<br>R: AGGATGGCAACCCATCACTG	This study
Abaecin	F: CAGCATTCGCATACGTACCA<br>R: GACCAGGAAACGTTGGAAAC	[6]
Apisimin	F: TGAGCAAAATCGTTGCTGTC<br>R: AACGACATCCACGTTCGATT	[6]
Defensin1	F: TGCGCTGCTAACTGTCTCAG<br>R: AATGGCACTTAACCGAAACG	[6]
Defensin2	F: GCTGCTACCACTACGACATC<br>R: CAACTACCGCCTTTACGTCG	[6]

Vittelogenin	F: ACGTAATAAATGCCGCCAAG<br>R: TGCATGTTGCTCTCCAACTC	This study

GAPDH: glyceraldehyde 3-phosphate dehydrogenase; MGST: microsomal glutathione s-transferase; eIF: eukaryotic initiation factors; PRM: protamine; RP: ribosomal proteins; RPL: ribosomal proteins of the large subunit; RPS: ribosomal proteins of the small subunit; SRC-C: scavenger receptor class C; TARBP: TAR RNA-binding protein; TBP-af: TATA box binding protein - associated factor; TRBP: TAR RNA-binding protein; AChE: Acetylcholinesterase; CYP: cytochrome P

[1] Cunha AD, Nascimento AM, Guidugli KR, Simoes ZLP, Bitondi MMG. (2005) Molecular cloning and expression of a hexamerin cDNA from the Honey bee, *Apis mellifera*. *J Insect Physiol* 51:1135–47.

[2] Scharlaken B, de Graaf DC, Goossens K, Brunain M, Peelman LJ, Jacobs FJ (2008) Reference gene selection for insect expression studies using quantitative real-time PCR: The head of the Honey bee, *Apis mellifera*, after a bacterial challenge. *J Insect Science* 8: 33

[3] Fisher P, Grozinger C.M. (2008) Pheromonal regulation of starvation resistance in Honey bee workers (Apis mellifera). *Naturwissenschaften* 95:723-729

[4] Cornman RS, Tarpy DR, Chen Y, Jeffreys L, Lopez D Pettis JS, Vanengelsdorp D, Evans JD (2012) Pathogen webs in collapsing Honey bee colonies. *PLoS One* 7:e43562

[5] Liao Z, Jia Q, Li F, Han Z (2010) Identification of two piwi genes and their expression profile in Honey bee, *Apis mellifera*. *Arch Insect Biochem Physiol* 74:91–102.

[6] Evans J (2006) Beepath: an ordered quantitative-PCR array for exploring Honey bee immunity and disease. *Journal of Invertebrate Pathology* 93:135-139.

[7] Lourenço AP, Mackert A, Cristino AS, Simões ZLP (2008) Validation of reference genes for gene expression studies in the Honey bee, Apis mellifera, by quantitative real-time RT-PCR. *Apidologie* 39:372–385.
